# Supplementary material for: Orbital-selective band engineering realizes high zT in p-type Ru2Ti1−xHfxSi full-Heusler thermoelectrics
Source: Nat Commun. 2026 Mar 9;17:2878. doi: 10.1038/s41467-026-69799-x (PMC13022361; doi:10.1038/s41467-026-69799-x)
Supplement: Supplementary file 1 — Supplementary Information [file 41467_2026_69799_MOESM1_ESM.pdf]

Supplementary Information for "Orbital-selective band engineering realizes high *zT* in *p*-type $Ru_2Ti_{1\text{-}x}Hf_xSi$ full-Heusler thermoelectrics"

Fabian Garmroudi[1,*,†], Illia Serhiienko[2,*], Michael Parzer[3], Andrej Pustogow[3], Raimund Podloucky[4], Takao Mori[2,5], Ernst Bauer[3,†]

[1]Materials Physics Applications – Quantum, Los Alamos National Laboratory, 87545 Los Alamos, New Mexico, USA.[2]Research Center for Materials Nanoarchitectonics (MANA), National Institute for Materials Science (NIMS), Tsukuba 305-0044, Japan. [3]Institute of Solid State Physics, TU Wien, Vienna A-1040, Austria. [4]Institute of Materials Chemistry, Universität Wien, Vienna A-1090, Austria. [5]Graduate School of Pure and Applied Sciences, University of Tsukuba, Tsukuba 305-8573, Japan.

* These authors contributed equally.

† Corresponding authors.

fgarmroudi@lanl.gov, bauer@ifp.tuwien.ac.at

1 SUPPORTING FIGURES AND TABLES

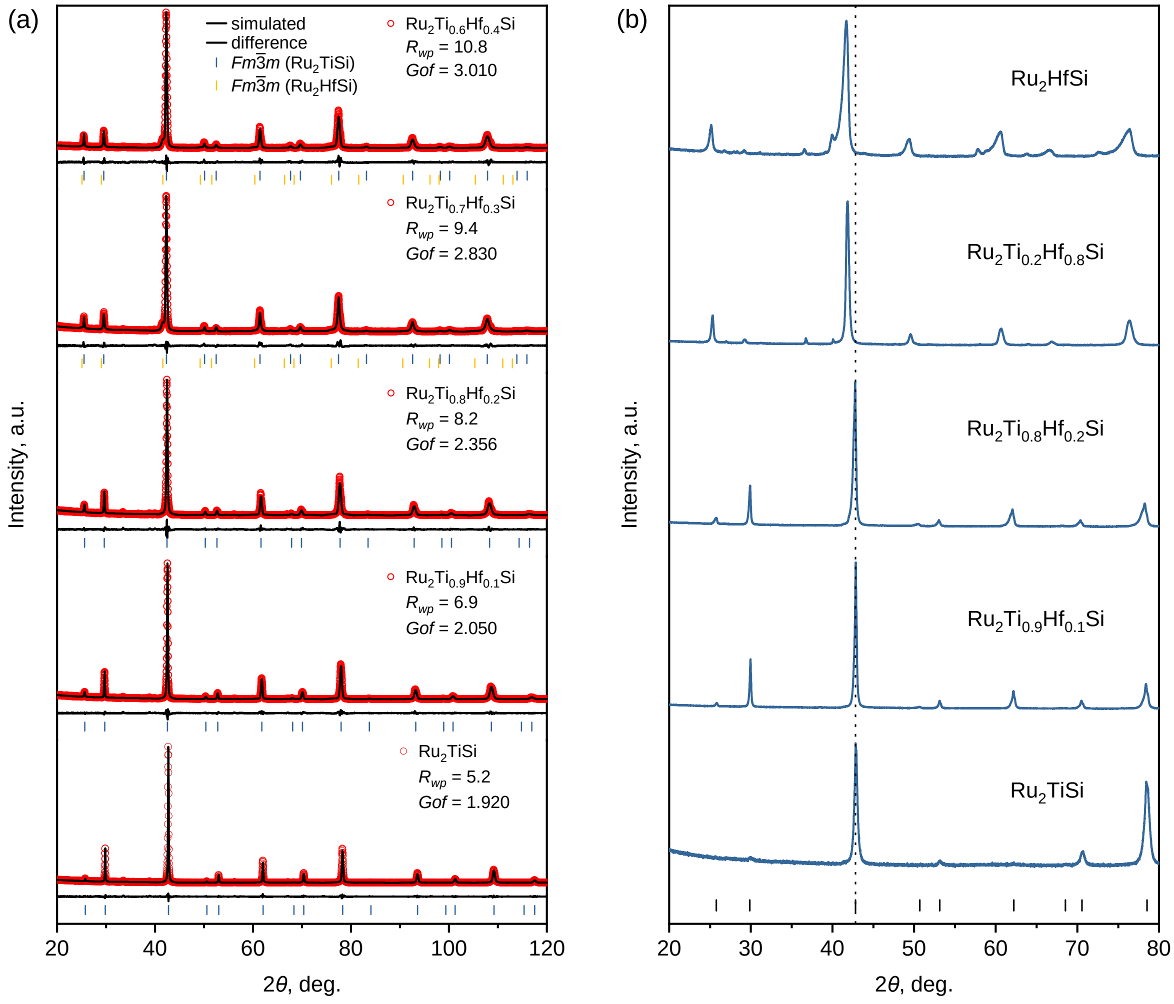

Figure S1 X-ray powder diffraction patterns (a) obtained at NIMS and (b) obtained at TU Wien. Black solid lines in panel (a) are Rietveld refinements with corresponding parameters listed in Table S1.

Table S1 Crystal structure refinement results of $Ru_2Ti_{1-x}Hf_xSi$ full-Heusler alloys obtained at NIMS, including lattice parameter a, isotropic atomic displacement parameters B_{ISO}, site occupancy factor (SOF), phase fractions (wt.%), weighted profile R-factor (R_{wp}), and profile R-factor (R_p)

Sample		Ru_2TiSi	$Ru_2Ti_{0.9}Hf_{0.1}Si$	$Ru_2Ti_{0.8}Hf_{0.2}Si$	$Ru_2Ti_{0.7}Hf_{0.3}Si$	$Ru_2Ti_{0.6}Hf_{0.4}Si$
Diffractometer		SmartLab				
Space group		$Fm\bar{3}m$				
2θ and θ/γ(max)		157 / 0.636				
a, Å		5.97624(3)	5.99394(3)	6.00933(9)	6.0250(1)	6.02436(8)
B_{ISO}	4a (Ti)	0.50(2)	0.68(13)	0.44(5)	0.18(3)	0.19(10)
	4b (Si)	1.1(1)	0.95(9)	0.93(8)	0.72(6)	0.9(4)
	4c (Ru)	0.79(4)	0.42(5)	0.33(3)	0.25(2)	0.35(7)
SOF	4a (Ti-Hf)	1	0.91(2)-0.09(2)	0.81(2)-0.19(2)	0.76(2)-0.24(2)	0.74(2)-0.26(2)
	4b (Si)	1	1	1	1	1
	4c (Ru)	0.98(1)	0.98(1)	1	0.99(3)	1
Main phase, wt.%		100	100	100	94.7(8)	91.9(7)
Secondary phase, wt.%		0	0	0	5.3(8)	8.1(7)
R_{wp}, %		5.2	6.9	8.2	9.4	10.8
R_p, %		4.9	6.5	5.8	9.2	11.5
FWHM		0.188(2)	0.256(2)	0.289(2)	0.350(3)	0.349(3)
ρ_{calc}, g/cm^3		8.65	8.98	9.31	9.64	10.04
ρ_{exp}, g/cm^3		8.17	8.76	9.04	9.41	9.8

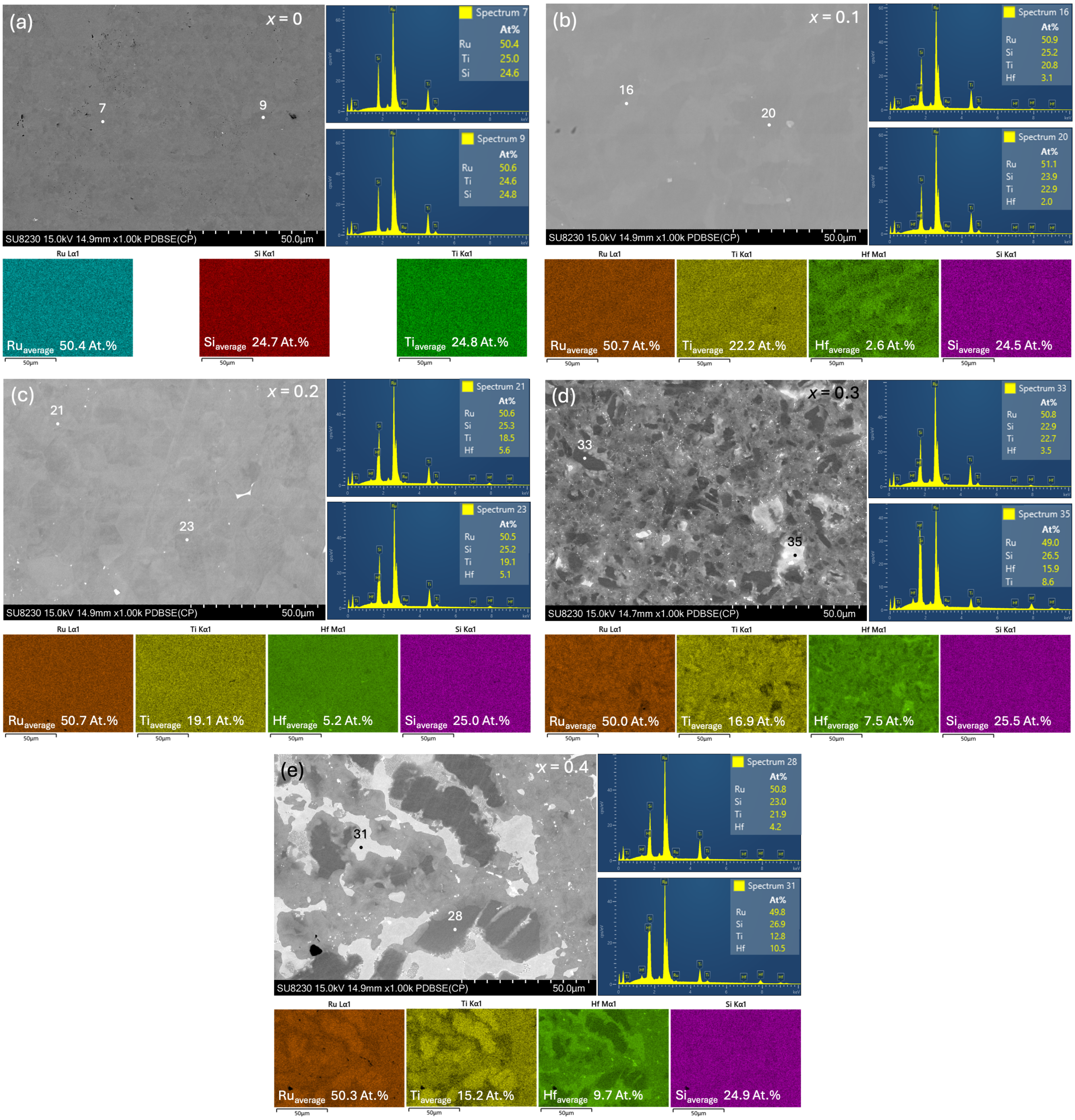

Figure S2 SEM micrographs of $Ru_2Ti_{1-x}Hf_xSi$ with $x = 0$, 0.1, 0.2, 0.3, and 0.4, alongside with EDX point spectra and element mapping.

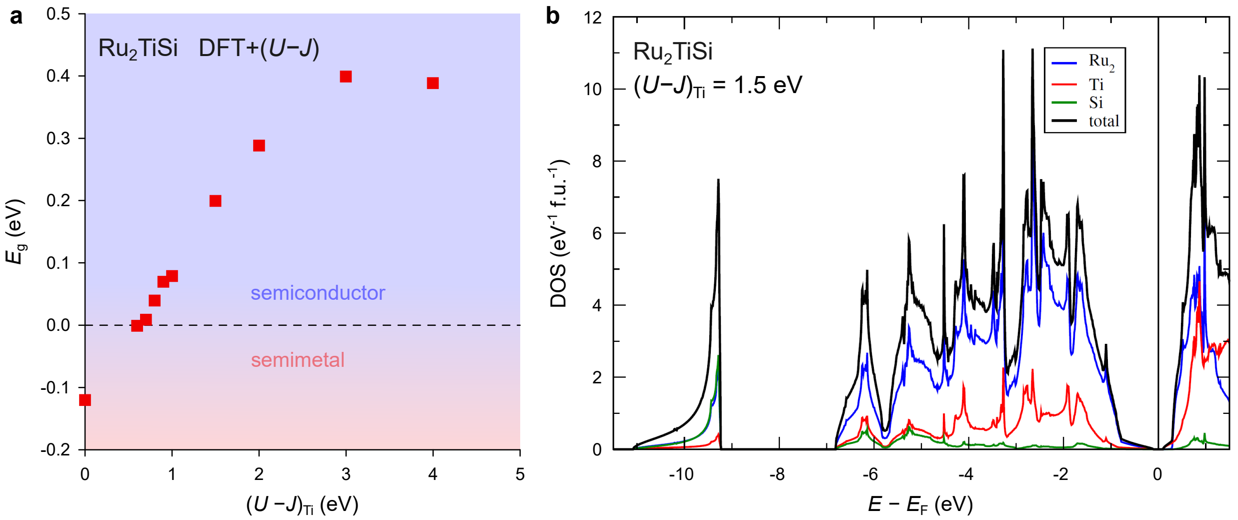

Figure S3 a, Band gap as a function of the effective on-site Coulomb interaction $(U-J)_{\mathrm{Ti}}$ for the Ti $3d$ states in Ru_2TiSi from DFT$+(U-J)$ calculations. The band gap increases as a function of $(U-J)_{\mathrm{Ti}}$ and a semimetal-semiconductor crossover occurs at $(U-J)_{\mathrm{Ti}} \approx 0.6$ eV. Values of around $(U-J)_{\mathrm{Ti}} \approx 1.5$ eV produce the best agreement with the experimental Seebeck coefficient and transport-derived band gap. **b**, Density of states from DFT$+(U-J)$ calculations corresponding to the recommended value $(U-J)_{\mathrm{Ti}} \approx 1.5$ eV.

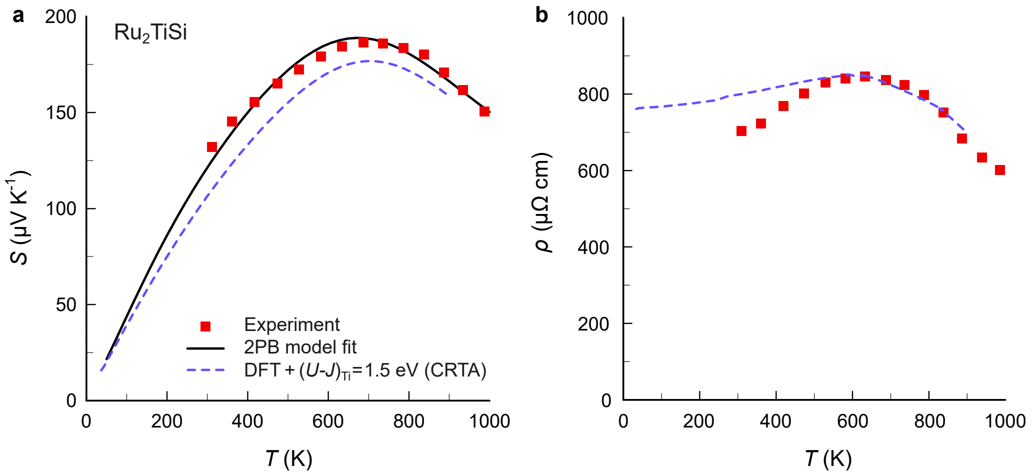

Figure S4 a, Temperature-dependent Seebeck coefficient of undoped Ru_2TiSi and comparison to theoretical calculations. A least-squares fit employing a two-parabolic band model can describe the experimental data very well. Transport calculations within the constant relaxation time approximation derived from the DFT band structure can only describe the experimental data when an additional effective on-site Coulomb interaction $(U-J)_{\mathrm{Ti}} \approx 1.5\,$eV for the Ti $3d$ states is taken into consideration. **b**, Temperature-dependent electrical resistivity. The constant relaxation time was chosen such that the calculated resistivity is normalized to the maximum of the experimental $\rho(T)$ curve.

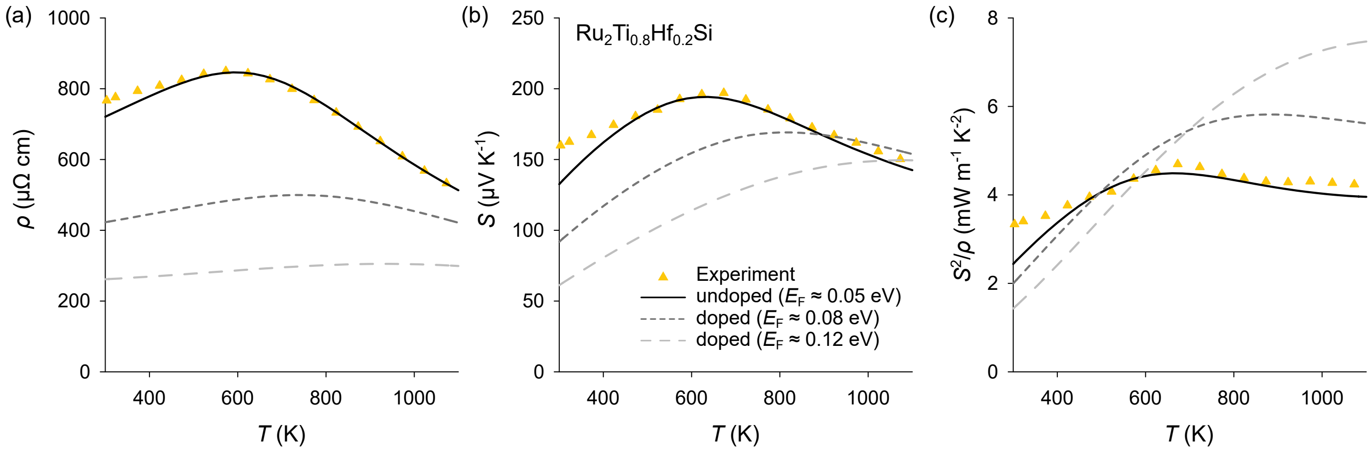

Figure S5 Temperature-dependent modeling of electronic transport properties of $Ru_2Ti_{0.8}Hf_{0.2}Si$. (a) Electrical resistivity, (b) Seebeck coefficient and (c) power factor. Black solid lines were obtained by simultaneously fitting $\rho(T)$ and $S(T)$, yielding remarkable agreement with experimental data. Grey dashed lines represent predictions for different Fermi level positions, showing that additional co-doping is required to further enhance the power factor of $Ru_2Ti_{0.8}Hf_{0.2}Si$.

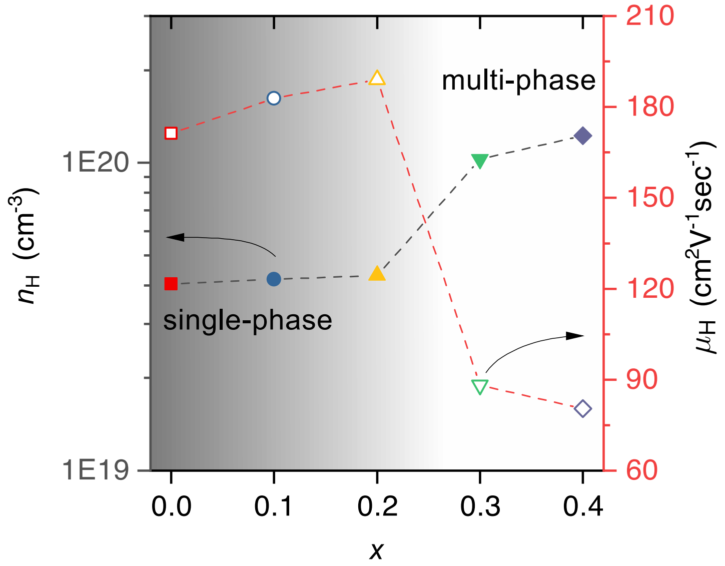

Figure S6 Hall carrier concentration and Hall mobility of Hf-substituted $Ru_2Ti_{1-x}Hf_xSi$ ($x = 0.1, 0.2, 0.3$ and 0.4) obtained at 300 K. In the single-phase regime ($x \leq 0.2$), the carrier concentration stays roughly constant, as expected from an isovalent substitution. Surprisingly, the Hall mobility does not decrease and even slightly increases, consistent with the evolution of the weighted mobility.
